# mRNA Vaccine Protects against Zika Virus

**DOI:** 10.3390/vaccines9121464

**Published:** 2021-12-10

**Authors:** Lex G. Medina-Magües, Janina Gergen, Edith Jasny, Benjamin Petsch, Jaime Lopera-Madrid, Emily S. Medina-Magües, Cristhian Salas-Quinchucua, Jorge E. Osorio

**Affiliations:** 1 Department of Pathobiological Sciences, School of Veterinary Medicine, University of Wisconsin, Madison, WI 53706, USA; loperamadrid@wisc.edu (J.L.-M.); esrussell@wisc.edu (E.S.M.-M.); cristhian.salas@wisc.edu (C.S.-Q.); 2 CureVac AG, Friedrich-Miescher-Straße 15, 72076 Tübingen, Germany; janina.gergen@curevac.com (J.G.); edith.jasny@curevac.com (E.J.); benjamin.petsch@curevac.com (B.P.)

**Keywords:** AG129, challenge, flavivirus, lipid nanoparticles, mice, mRNA vaccines, RNActive^®^, Zika vaccines, Zika virus, ZIKV

## Abstract

Zika virus (ZIKV), a mosquito-borne flavivirus, has recently triggered global concern due to severe health complications. In 2015, a large ZIKV outbreak occurred in the Americas and established a link between ZIKV and microcephaly in newborn babies, spontaneous abortion, persistent viremia, and Guillain–Barré syndrome. While antivirals are being developed and prevention strategies focus on vector control, a safe and effective Zika vaccine remains unavailable. Messenger RNA (mRNA) vaccine technology has arisen as a flexible, simplified, and fast vaccine production platform. Here, we report on an mRNA vaccine candidate that encodes the pre-membrane and envelope (prM–E) glycoproteins of ZIKV strain Brazil SPH2015 and is encapsulated in lipid nanoparticles (LNPs). Our ZIKV prM–E mRNA-LNP vaccine candidate induced antibody responses that protected in AG129 mice deficient in interferon (IFN) alpha/beta/gamma (IFN-α/β/γ) receptors. Notably, a single administration of ZIKV prM–E mRNA-LNP protected against a lethal dose of ZIKV, while a two-dose strategy induced strong protective immunity. E-specific double-positive IFN-γ and TNF-α T-cells were induced in BALB/c mice after immunizations with a two-dose strategy. With the success of mRNA vaccine technology in facing the coronavirus (COVID-19) pandemic, our data support the development of prM–E RNActive^®^ as a promising mRNA vaccine against Zika to counter future epidemics.

## 1. Introduction

Emerging zoonotic infectious diseases present a significant global health burden. While the first case of ZIKV infection in humans was detected in Nigeria in 1952 [1], this arbovirus, transmitted from *Aedes* spp. mosquitoes, was unremarkable for decades until outbreaks occurred between 2007 to 2017 on Yap Island [2], French Polynesia [3], and the Americas [4]. After the 2015 outbreak in Brazil, where the first case of autochthonous transmission of ZIKV was detected [5], efforts began to prioritize ZIKV surveillance and the development of Zika vaccines. The global interest was primarily due to the detrimental fetal outcomes in pregnant women infected with ZIKV in Brazil and other countries around the world [6].

Neutralizing antibodies (nAbs) are key mediators of protection against flavivirus infections and have been correlated with efficacy for Zika vaccines [7,8,9]. Although global interest in Zika has led to a variety of vaccine candidates, to date, there is no licensed vaccine for the disease. Vaccine platforms targeting the viral envelope protein (E), responsible for mediating cell fusion, and the pre-membrane protein (prM) induce high levels of nAbs. Potential vaccine platforms against Zika need to be safely administered to pregnant women, the most vulnerable population at risk for ZIKV infection. Since pregnant women have been excluded from clinical trials thus far, little information is available on the safety of Zika vaccine candidates in this specific population.

Although vaccine platforms against ZIKV are broad-reaching, just two vaccine candidates utilizing mRNA technology have been investigated in clinical trials [10]. Demonstrated in the global pandemic of COVID-19, mRNA vaccine technology is a safe and effective means to stimulate protective immune responses. In this study, we aimed to investigate a Zika vaccine candidate using LNP encapsulated mRNA technology for further clinical development. Unlike current vaccine candidate platforms, mRNA vaccines do not pose a risk of infection and insertional mutagenesis, and they avoid the risk of anti-vector immunity, allowing for repeated administration [11]. Different modifications and delivery methods allow the regulation of in vivo half-life and immunogenicity and increase the efficiency of mRNA delivery, uptake, and expression in target cells [11]. For a Zika vaccine that can be administered safely to different age groups and pregnant women, mRNA vaccines might address the theoretical risks associated with live vaccine use.

Here, we evaluated the efficacy of an mRNA vaccine candidate (ZIKV prM–E mRNA-LNP) in an AG129 mouse model. We demonstrated that a single dose of ZIKV prM–E mRNA-LNP protected animals after lethal ZIKV challenge infection. Compared with placebo, vaccinated animals did not develop clinical signs or body weight loss, and they showed reduced viral loads. Remarkably, in this model, a two-dose strategy of ZIKV prM–E mRNA-LNP vaccine induced strong immunity. Lastly, vaccination of BALB/c mice followed by T-cell analysis of the isolated splenocytes demonstrated antigen-specific CD4^+^ and CD8^+^ T-cell responses. This study paves the way for further preclinical and clinical development of the ZIKV prM–E mRNA-LNP vaccine candidate.

## 2. Materials and Methods

### 2.1. Production of the mRNA Vaccines

**Figure 1:**
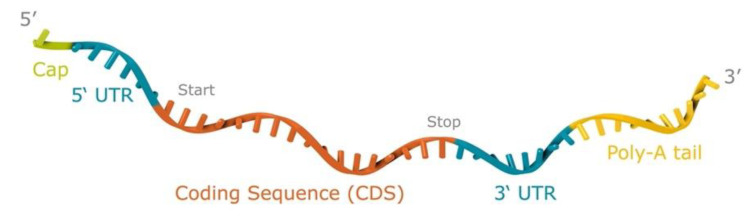
Schematic outline of the ZIKV prM–E mRNA-LNP vaccine candidate construct. Reprinted with permission from Springer Nature Customer Service Center GmbH: Springer Nature [Gergen J., Petsch B. (2020) mRNA-Based Vaccines and Mode of Action. In: Current Topics in Microbiology and Immunology. Springer, Berlin, Heidelberg. https://doi.org/10.1007/82_2020_230, accessed on 6 December 2021].

The mRNA vaccine is based on the RNActive^®^ platform (claimed and described in, e.g., WO2002098443 and WO2012019780) and comprises a 5′ Cap1 structure (CleanCap™), GC-enriched open reading frame (ORF), 3′ UTR, and polyA tail, whereas it does not include chemically modified nucleosides (Figure 1). LNP encapsulation of mRNA was performed by Acuitas Therapeutics (Vancouver, Canada). The LNPs used in this study are particles of ionizable amino lipids, phospholipids, cholesterol, and PEGylated lipids. The mRNA encodes prM–E of ZIKV (strain Brazil-SPH2015) with the C-terminal stem region of the envelope protein substituted by the respective stem region derived from the envelope protein of Japanese encephalitis virus.

### 2.2. Cells and Viruses

Vero (ATCC: CCL-81) and C6/36 cells (ATCC: CRL-1660) were cultured in Dulbecco’s modified Eagle medium (DMEM; Corning, VA), 10% fetal bovine serum (FBS), and antibiotics incubated with 5% CO₂ at 37 °C or 28 °C, respectively. H/PF/2013 ZIKV strain (GenBank: KJ776791) was amplified by inoculating a confluent monolayer of C6/36 cells with an MOI of 0.01 and was stored at −80 °C until use. Viral titer was determined by plaque assay using Vero cells and expressed as plaque-forming units per milliliter (PFU/mL) following the procedure described previously [12].

### 2.3. Animal Studies

BALB/c mice (8–12 weeks of age) were provided and handled by Preclinics Gesellschaft für präklinische Forschung mbH (Potsdam, Germany). BALB/c mouse experiments were conducted under German laws and guidelines for animal protection and appropriate local and national approvals. Mice were injected intramuscularly (I.M.) on days 0 and 21 with 5 µg of the ZIKV prM–E mRNA-LNP vaccine candidate or 0.9% NaCl buffer as the negative control. Serum samples were collected on days 21 and 35 for IgG1 and IgG2a analysis. The study was terminated on day 35, and the mouse splenocytes were isolated and stored as a single cell suspension at −80 °C until use.

AG129 mice (IFNAR^−/−^, IFNGR^−/−^) of the 129/SvEv genetic background were obtained from B&K Universal Limited (Hull, England) and were bred in the pathogen-free animal facilities of the University of Wisconsin-Madison (UW-Madison), School of Veterinary Medicine, in accordance with UW husbandry protocols (# G005519-R01-A01). Eight mice (4–6 weeks old, mixed sex) were used per group. The ZIKV prM–E mRNA-LNP vaccine was administered I.M. into the *M. tibialis*. One treatment group received two immunizations 14 days apart with 5 µg of ZIKV prM–E mRNA-LNP vaccine. A second group received one vaccination of 10 µg of ZIKV prM–E mRNA-LNP vaccine. A positive control group received two immunizations with 2 µg of ZIKV VLP (ZIKVLP) vaccine candidate formulated with aluminum hydroxide developed at UW-Madison and described previously [13], while the negative control group received 0.9% NaCl buffer. After vaccination, the animals were monitored for 44 days. Maxillary vein blood draws were performed pre-boost (day 14), pre-challenge (day 28), and 16 days after challenge (day 44) to collect serum for analysis of nAbs. Mice were bled 3 days after the challenge to determine viral load (day 31). Additional blood draws on day 21 were pooled with serum from day 28 for a passive transfer study. Blood was collected using Microvette^Ⓡ^ 500 Z-Gel (VWR, Cat. No. 20.1344), kept at room temperature (RT) for 15 min, and centrifuged at 10,000× *g* at 20 °C for 5 min. Serum was aliquoted and stored at −80 °C until use. Vaccinated mice were challenged I.M. with a dose of 100 PFU of H/PF/2013 ZIKV strain in 30 µL of phosphate-buffered saline (PBS). Mice that were moribund or lost more than 20% of the initial body weight after the challenge were humanely euthanized. A passive transfer study was carried out using four groups of 9–10 weeks old male mice (four per group). The mice were injected by intraperitoneal injection (I.P.) with 300 µL of pooled serum collected on days 21 and 28 of the vaccination study. Passively transferred mice were challenged I.M. with 10 PFU of H/PF/2013 ZIKV strain administered in 30 µL of PBS. Blood samples collected 12 h and 21 days after passive transfer were used to measure nAbs, and those collected on day 3 post-challenge were used to measure viral load.

### 2.4. Enzyme-Linked Immunosorbent Assay (ELISA)

Anti-E IgG1 and IgG2 antibodies were analyzed by in-house ELISA; 96-well Maxisorp ELISA plates (black) were coated with 2 µg/mL rec ZIKV E protein (Aalto Bio Reagents, Cat. No. AZ 6312) overnight. Plates were washed (1× PBS, 0.05% Tween-20) and blocked at 4 °C for 2 h with 1% milk in PBS/0.05% Tween-20. Serum samples were added in serial dilution (1:5) with a 1:10 starting dilution and incubated at RT for 2–4 h. After additional washing, plates were incubated with anti-IgG1 (1:300, BD Pharmingen, Cat. No. 550331) or anti-IgG2 (1:300, BD Pharmingen Cat. No. 550332) antibody in blocking buffer for 1 h. For detection, HPR-Streptavidin (1:1000, BD Pharmingen, Cat. No. 554066) was added for 30 min. Finally, plates were washed five times before addition of the Amplex^®^ UltraRed reagent (1:200, Invitrogen, Cat. No. A36066) with H_2_O_2_ (1:2000). Fluorescence was detected after 45–60 min.

### 2.5. T-Cell Analysis

The induction of antigen-specific T-cells was analyzed upon peptide stimulation using intracellular cytokine staining (ICS). Two million splenocytes per well (in 200 µL) were stimulated for 1 h at 37 °C using ZIKV PepMix Pool 2 (JPT, Cat. No. 29927) at 2 µg/mL. To inhibit the cytokine secretion, splenocytes were treated with Golgi Plug (BD Biosciences, Cat. No. 51-2301 ZK) in a dilution of 1:200 (50 µL) at 37 °C for 5–6 h. Afterward, cells were washed and kept in medium at 4 °C overnight. The next day, splenocytes were washed twice in PBS and stained with AquaDye (Invitrogen, Cat. No. L34957) solution at 4 °C for 30 min. After an additional washing step in FACS buffer (PBS with 0.5% bovine serum albumin), cells were surface stained for Thy1.2, CD4, and CD8 and incubated with FcƴR-block for 30 min at 4 °C in FACS buffer. Subsequently, cells were washed and fixed using Cytofix/Cytoperm (BD Biosciences) according to the manufacturer’s instructions. Finally, intracellular cytokine staining for induction of interferon-gamma (IFN-γ) and tumor necrosis factor-alpha (TNF-α) was done at 4 °C for 30 min. For the FACS analysis, splenocytes were resuspended in PFEA buffer (PBS, 2% FBS, 2 mM EDTA, and 0.01% azide). Splenocytes were analyzed on a Canto II flow cytometer (BD Biosciences, San Jose, CA, USA). Flow cytometry data were analyzed using FlowJo software (Tree Star, Inc., Ashland, OR, USA). The following antibodies were used for flow cytometry analysis: anti-Thy1.2 FITC (clone 53-2.1; Biolegend, Cat. No. 14304), anti-CD4 V450 (clone RM4-5; BD Biosciences, Cat. No. 560468), anti-CD8a APC-H7 (clone 53-6.7; BD Biosciences, Cat. No. 560182), anti-IFN-γ APC (clone XMG1.2, BD Biosciences, Cat. No. 554413), and anti-TNF-α PE (clone MP6-XT22, eBioscience, Cat. No. 12-7321-82).

### 2.6. Plaque Reduction Neutralization Test (PRNT)

A PRNT assay was performed using fourfold serial dilutions (starting 1:20) of each serum sample to test neutralization of H/PF/2013 ZIKV (200 PFUs) using Vero cells. Briefly, 96-well plates were seeded and used 24 h after reaching 90% confluency. Serum samples were heat-inactivated by incubation at 56 °C for 30 min, diluted in DMEM using fourfold dilutions, combined with equal volume with 200 PFUs of H/PF/2013 ZIKV, and incubated for 1 h at 37 °C and 5% CO₂. Vero cells were then infected with 50 µL of the inoculum (serum–virus mix) and incubated for 2 h. Following incubation, the inoculum was removed from the plate, and 150 µL of overlay solution (DMEM with 1.5% carboxymethyl cellulose and 2% FBS) was added per well and incubated at 37 °C and 5% CO₂. The overlay was discarded 36 h post infection, and 200 µL of fixing buffer (3.7% paraformaldehyde solution in PBS) was added per well and incubated for 30 min at RT. The immunoassay used 1:2000 dilution of the ZIKV hyperimmune mouse ascitic fluid (donated by University of Texas Medical Branch (UTMB)) in blocking buffer (PBS with 0.05% Tween-20 and 5% powdered milk), followed by 1:5000 diluted horseradish peroxidase-conjugated goat anti-mouse IgG (H + L) (Sigma-Aldrich, Cat. No. AP308P) as a secondary antibody and developed with Chromogen/Peroxide substrate. The plates were scanned in the ELISPOT plate reader (ImmunoSPOT-Cellular Technology, Cleveland, OH, USA), and the number of replication foci was counted using the counting function software. The neutralization percentage was calculated as indicated previously [13]. Neutralization percentages (Nx) were calculated per sample, replicate, and dilution as follows: Nx = 100 − (100 × (A/control)), where A corresponds to the number of counted foci in each well, and control is the geometric mean of foci counted from wells treated with cells and virus only (no sample). The log (1/dilution) vs. the mean values of Nx per sample were plotted and fitted to a sigmoidal dose–response curve, and PRNT_50_ values for each sample were interpolated using GraphPad Prism software.

### 2.7. Viral Load

RNA was extracted from 20 µL of serum collected 3 days after the challenge, using TRI Reagent^®^ BD (Molecular Research Center, Cat. No. TB 126) according to the manufacturer’s protocol. Viral RNA load was quantified using quantitative reverse-transcription polymerase chain reaction analysis (qRT-PCR), using the primers and probe described previously [14] (ZIKV 1086, ZIKV 1162c, and ZIKV 1107-FAM-ZEN). The qRT-PCR was performed using the iTaq™ Universal Probes One-Step Kit (Bio-Rad, Cat. No. 1725141) on an iCycler iQ™ Real-Time PCR Detection System (Bio-Rad, Hercules, CA, USA) and followed the manufacturer’s recommended protocol. RNA standard samples were prepared using in vitro transcribed RNA produced in house. In brief, RNA was extracted from the ZIKV H/PF/2013 strain using TRIzol™ LS Reagent (Invitrogen, Cat. No. 10296028). The cDNA was synthesized using the random primers and SuperScript VILO (Invitrogen, Cat. No. 11754050). The amplification of the target region and the addition of the T7 promoter was done with the primer ZIKV 835 [14] (with a T7 promoter sequence incorporated) and the primer ZIKV 1162c using OneTaq^®^ Quick-Load^®^ 2× Master Mix with Standard Buffer (NEB, Cat. No. M0486S). RNA was synthesized using MEGAscript T7 (Invitrogen, Cat. No. AM1334), TURBO DNAse-treated, and purified with UltraPure™ phenol–chloroform–isoamyl alcohol (25:24:1) (Invitrogen, Cat. No. 15593-031); the concentration was measured. The standard curve consisted of 10-fold dilutions of in vitro transcribed RNA, with the lowest copies per reaction being 100.

### 2.8. Statistical Analyses

Significance of the survival rates was assessed by a log-rank test due to the right-skewed and censored nature of the data. The viral load was assessed using a one-way analysis of variance (ANOVA) with Tukey’s multiple comparison test, while ELISA and PRNT_50_ titers were assessed by two-way ANOVA, and the differences in means between studies were determined using Šidák’s multiple comparison test. These tests were performed after the data were log-transformed and assessed for normality using a QQ plot. After population normality was confirmed using a QQ plot, Levene’s test was performed to ensure equality of variances. For the T-cell analysis, a Mann–Whitney U test was implemented. All data were analyzed with GraphPad Prism version 9.1.1 software for Mac (GraphPad Software, San Diego, CA, USA, www.graphpad.com, accessed on 6 December 2021). A value of *p* < 0.05 was considered statistically significant (* *p* < 0.05, ** *p* < 0.01, *** *p* < 0.001, **** *p* < 0.0001, ns *=* not significant).

## 3. Results

### 3.1. ZIKV prM–E mRNA-LNP Vaccine Candidate Induces E Protein-Specific IgG1 and IgG2a Isotype Antibodies and T-Cell Responses in BALB/c Mice

**Figure 2:**
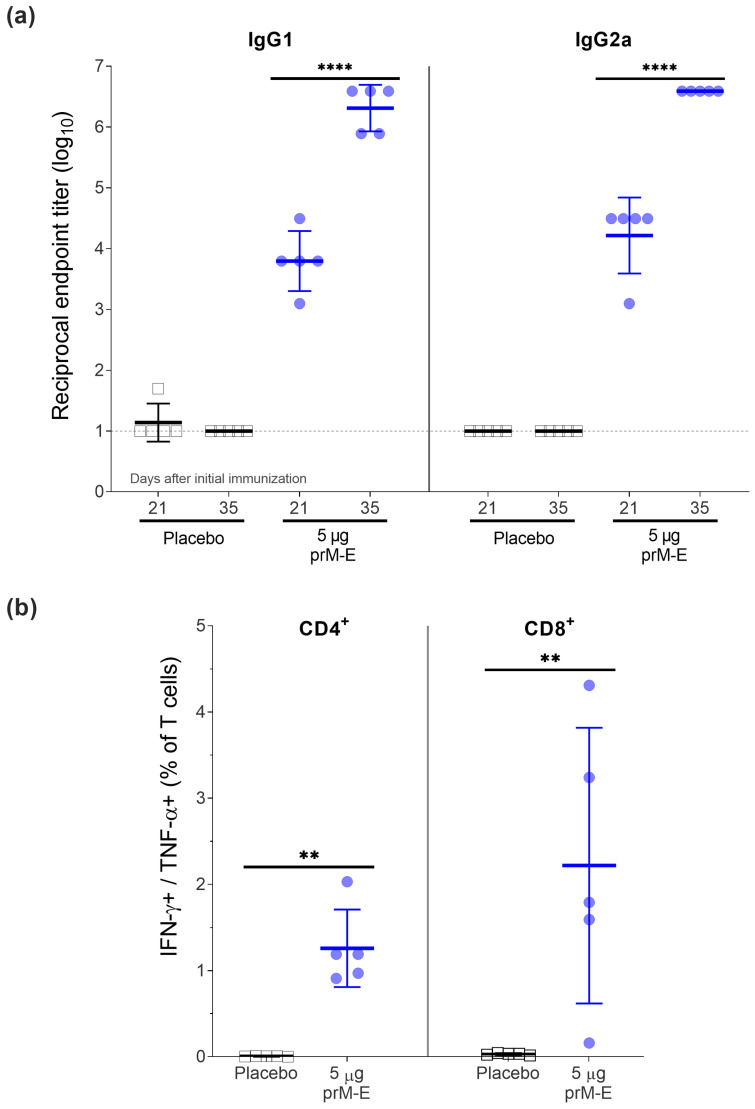
ZIKV prM–E mRNA-LNP vaccine candidate induces high titers of IgG1 and IgG2a antibodies and T-cell responses in BALB/c mice (*n =* 5 per group) that received two doses of 5 µg of ZIKV prM–E (circle; blue) or placebo injection of NaCl buffer (open square; black) 21 days apart. Mice were euthanized for splenocyte isolation on day 35. (**a**) Profile of IgG1 and IgG2a antibody responses (geometric mean ± geometric SD) measured in serum before the second immunization (day 21) and before euthanasia (day 35). (**b**) Frequency of IFN-γ+/TNF-α+ on CD4^+^ and CD8^+^ T-cell subsets measured by intracellular cytokine staining 2 weeks after the second immunization (mean ± SD). Raw data used for the figures are available in the Supplementary Materials. Dashed line shows the limit of detection; ** *p* < 0.01, **** *p* < 0.0001. Two-way ANOVA followed by Šidák’s multiple comparisons test was performed for the IgG1 and IgG2a antibody titers. These tests were performed after the data were log-transformed and assessed for normality using a QQ plot. For the T-cell analysis, a Mann–Whitney U test was implemented. All tests were performed using GraphPad Prism.

An optimal Zika vaccine candidate should induce a high antibody titer against the E protein and a strong cellular immune response. To assess the immune response induced by the ZIKV prM–E mRNA-LNP vaccine candidate, we vaccinated 8–12 weeks old BALB/c mice twice (day 0 and 21; I.M.). The humoral immune response was analyzed by IgG1 and IgG2a ELISA after the first and second immunization (Figure 2a). IgG1 and IgG2a titers were similar at both time points. The second vaccination significantly increased the geometric mean titer (GMT) by around 2 log_10_. Additionally, T-cell responses were determined on day 35 by ICS using flow cytometry. Compared to the placebo group, most vaccinated mice elicited robust ZIKV E-specific CD4^+^ and CD8^+^ T-cell responses by measuring the intracellular production of IFN-γ and TNF-α (Figure 2b).

This data demonstrated that a two-dose regimen of the ZIKV prM–E mRNA-LNP vaccine candidate induced both strong ZIKV E-specific IgG antibody responses (IgG1 and IgG2a) and antigen-specific T-cell responses.

### 3.2. ZIKV prM–E mRNA-LNP Vaccine Candidate Protected AG129 Mice from Challenge Infection with a Lethal Dose of ZIKV

#### 3.2.1. Vaccination and Challenge

**Figure 3:**
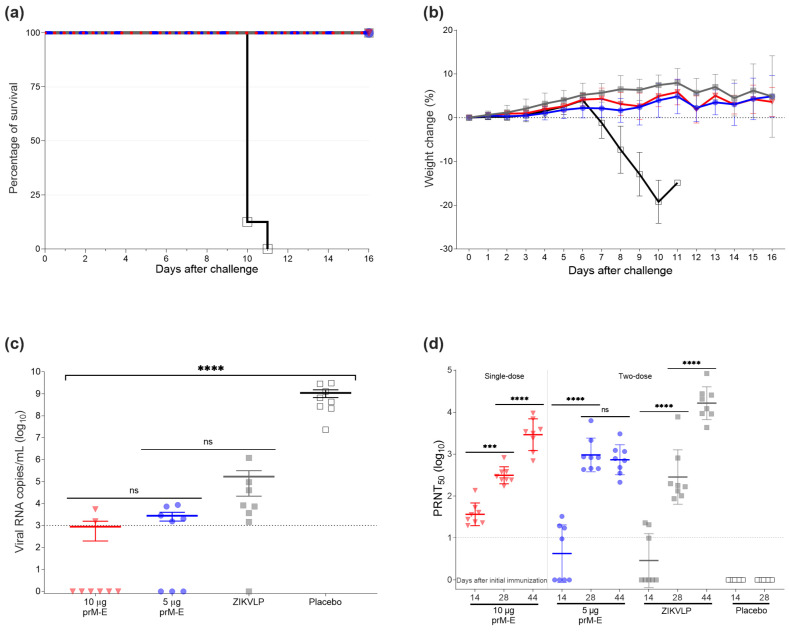
ZIKV prM–E mRNA-LNP vaccine protects against lethal I.M. ZIKV challenge in mice. (**a**) Survival curve of four groups of AG129 mice (*n = 8* per group) that received one single dose of 10 µg of ZIKV prM–E (inverted triangle; red), two doses of 5 µg of ZIKV prM–E (circle; blue), two doses of 2 µg ZIKVLP (close square; gray), or placebo injection (open square; black) 14 days apart. Four weeks later, on day 28, they were infected I.M. with 100 PFU of ZIKV. (**b**) Percentage body weight change (mean ± SD) of challenged animals over a period of 16 days post challenge. Mice were euthanized once they showed severe clinical signs of disease or lost 20% of body weight. A single placebo-immunized mouse was euthanized on day 11. (**c**) Viral load (mean ± SEM) measured by qRT-PCR 3 days after the challenge. (**d**) Neutralizing antibody titers (geometric mean ± geometric SD) measured in serum before second immunization (day 14) and challenge (day 28), and 16 days after challenge (day 44). Raw data used for the figures are available in the Supplementary Materials. Dashed line shows the limit of detection; *** *p* < 0.001, **** *p* < 0.0001, ns = not significant. One-way ANOVA followed by Tukey’s multiple comparisons test or two-way ANOVA followed by Šidák’s multiple comparisons test was performed using GraphPad Prism for viral load and neutralizing antibody titers, respectively. These tests were performed after the data were log-transformed and assessed for normality using a QQ plot.

To assess vaccine efficacy in AG129 mice, we vaccinated 4–6 weeks old mice with the ZIKV prM–E mRNA-LNP vaccine candidate. Animals were injected I.M. twice 14 days apart with 5 µg of the ZIKV prM–E mRNA-LNP vaccine candidate, 2 µg of the ZIKVLPs as a positive control, and NaCl buffer as a negative control, while 10 µg of the ZIKV prM–E mRNA-LNP vaccine was administered in a single dose. After vaccination, no noticeable inflammation at the injection site was observed. All ZIKV prM–E mRNA-LNP- and ZIKVLP-vaccinated mice survived the lethal challenge (*p* < 0.0001) compared to the placebo mice that reached humane endpoints (moribund or lost more than 20% of the initial body weight) 11 days after the challenge (Figure 3a). No body weight loss or clinical signs were observed for groups immunized with ZIKV prM–E mRNA-LNP or ZIKVLP, respectively (Figure 3b). For NaCl buffer-treated mice, clinical signs such as lethargy, paralysis, body weight loss, and hunched posture were the primary reasons for euthanasia. qRT-PCR identified a significant effect on viral load in serum collected 3 days after the challenge (F_3,28_ = 38.70, *p* < 0.0001) (Figure 3c). Compared to placebo-treated animals, all vaccinated mice showed substantially reduced viral RNA copies (*p* < 0.0001) (Figure 3c). The lowest viral load was found for both groups immunized with ZIKV prM–E mRNA-LNP vaccine with a mean of 8.9 × 10^2^ copies/mL for the single dose of 10 µg and 2.8 × 10^3^ copies/mL for the vaccine group that received 5 µg twice, followed by the ZIKVLP vaccine (1.7 × 10^5^ copies/mL). Before the second vaccination on day 14, the PRNT_50_ titer in mice vaccinated with 10 µg of ZIKV prM–E mRNA-LNP vaccine was higher than that in the ZIKVLP group (*p* = 0.0003) (Figure 3d). Furthermore, a dose dependency was observed for the ZIKV prM–E mRNA-LNP vaccine, showing a GMT of 10.3 for mice receiving 5 µg and a GMT of 45.0 for mice receiving 10 µg of mRNA (*p* = 0.0022). Before the challenge, all vaccinated groups showed similar PRNT_50_ titers. Interestingly, the GMT of the group immunized twice with 5 µg of ZIKV prM–E mRNA-LNP vaccine did not increase after challenge (*p* = 0.7370), while high PRNT_50_ titers were detected after challenge on day 44 for surviving mice in the remaining vaccine groups (*p* < 0.0001).

#### 3.2.2. Passive Transfer Protected Mice against ZIKV Lethal Challenge Infection

**Figure 4:**
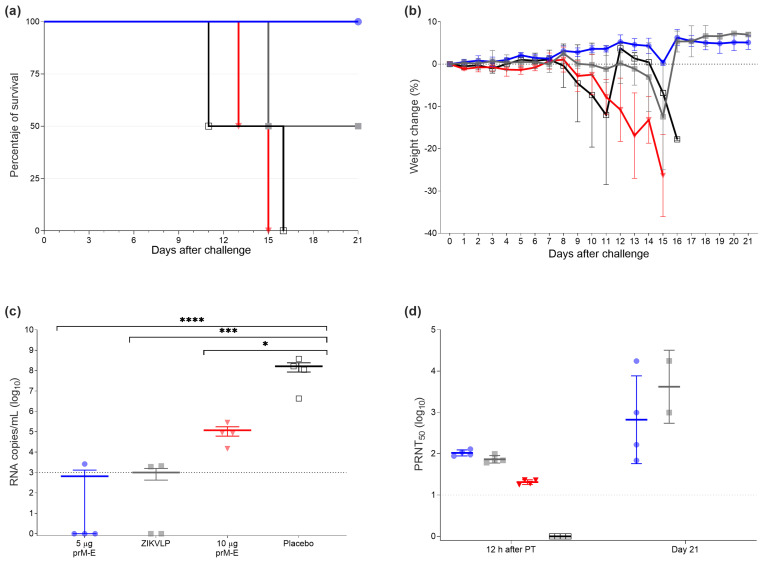
Passive transfer protected mice against lethal ZIKV challenge. (**a**) Survival curve of four groups of AG129 mice (*n =* 4 per group) which received immune sera via I.P. injection from mice vaccinated with one dose of 10 µg of ZIKV prM–E (inverted triangle; red), two doses of 5 µg of ZIKV prM–E (circle; blue), ZIKVLP (close square; gray), or placebo injection (open square; black). Twelve hours later, mice were challenged with 10 PFU of ZIKV. (**b**) Percentage of the body weight change (mean ± SD) of challenged animals over a period of 21 days after the challenge. Mice were euthanized once they showed severe clinical signs of disease or lost 20% body weight. (**c**) Viral load (mean ± SEM) measured by qRT-PCR 3 days after the challenge. (**d**) Neutralizing antibody titers (geometric mean ± geometric SD) measured in serum 12 h after passive immunization and at the end of the study (day 21). Raw data used for the figures are available in the Supplementary Materials. Dashed line shows the limit of detection; PT = passive transfer; * *p* < 0.05, *** *p* < 0.001, **** *p* < 0.0001. One-way ANOVA followed by Tukey’s multiple comparisons test was performed using GraphPad Prism for viral load and neutralizing antibody titers. These tests were performed after the data were log-transformed and assessed for normality using a QQ plot.

To assess the protection mediated via the humoral immune responses elicited by the ZIKV prM–E mRNA-LNP vaccine candidate, we performed a passive transfer experiment in naïve AG129 mice using pooled serum from vaccinated mice. In brief, pooled pre-challenge sera collected in the vaccination experiment on days 21 and 28 were injected via I.P. injection (300 µL per mouse). The GMTs for the pooled sera were 5982 for the group vaccinated twice with 5 µg ZIKV prM–E mRNA-LNP, 514 for the mice immunized once with 10 µg ZIKV prM–E mRNA-LNP, and 1861 for the ZIKVLP vaccine. Twelve hours after the passive immunization, mice were bled, challenged, and monitored daily for 3 weeks. All recipient mice of two immunizations with 5 µg of ZIKV prM–E mRNA-LNP vaccine survived the challenge (survival 4/4), followed by the mice which received the sera from the ZIKVLP immunized group (survival 2/4). Meanwhile, all recipient mice of the sera collected from the ZIKV prM–E mRNA-LNP10 µg immunized group and placebo groups died 15 and 16 days after the challenge, respectively (Figure 4a). Mice that received sera from animals vaccinated twice with 5 µg of ZIKV prM–E mRNA-LNP displayed no body weight loss or clinical signs (Figure 4b). There was a statistically significant effect detected on the viral load in serum collected 3 days after challenge (F_3,12_ = 21.80, *p* < 0.0001) (Figure 4c). The results for the viral load showed that, compared to placebo serum-transferred mice, the ZIKV prM–E mRNA-LNP 5 µg group vaccinated twice (*p* < 0.0001), ZIKVLP (*p* = 0.0002), and 10 µg group vaccinated once (*p* = 0.0418) serum-transferred mice showed substantially reduced viral RNA copies. No measurable viral RNA was detected for three mice that received serum from the ZIKV prM–E mRNA-LNP 5 µg group and two ZIKVLP sera-transferred mice. Consistent with the low viral RNA levels, high PRNT_50_ titers were also observed in the ZIKV prM–E mRNA-LNP 5 µg two-dose group (GMT = 105.1), ZIKVLP (GMT = 74.3), and ZIKV prM–E mRNA-LNP 10 µg single-dose group (GMT = 20.6) of serum-transferred mice at the time point of the challenge (Figure 4d).

## 4. Discussion

As a proof of principle, this study evaluated a highly efficacious Zika vaccine candidate using RNActive^®^ technology with low reactogenicity in mice. This mRNA vaccine encoded prM–E of ZIKV strain Brazil SPH2015 and induced nAbs that protected AG129 mice after a lethal challenge. AG129 mice have been extensively used as an animal model to demonstrate the efficacy of several flavivirus vaccines, including ZIKV. They are double-knockout for type I and II interferon receptors and consequently highly susceptible to ZIKV infection and pathogenicity [15]. The ZIKV mRNA vaccine formulation was safe as immunized animals did not show any post-vaccination side-effects. Furthermore, robust, functional antibody responses were induced in both mRNA vaccinated groups. Very low viral load was observed following the ZIKV challenge, with no morbidity and mortality. After challenge, animals vaccinated with two doses did not show an increase in nAb titers, suggesting induction of strong protective immunity. As nAbs play a significant role in the immune response to infection with ZIKV and other flaviviruses, the ability to induce strong immunity shows high promise that this vaccine candidate may induce protection in humans [16]. In the passive transfer study, we demonstrated that the ZIKV prM–E mRNA-LNP vaccine induced protective antibodies. The minimal antibody protective titer remains to be determined. The initial GMT of 5982 (from the ZIKV prM–E mRNA-LNP 5 µg vaccine pooled sera) was diluted in the circulation of recipient AG129 mice to levels ranging from 88 to 129 (GMT = 95). This level of circulating antibodies was sufficient to fully protect mice from the subsequent ZIKV challenge. Pooled sera (GMT = 1861) from the ZIKVLP-immunized group resulted in circulating nAb titers ranging from 60 and 98 (GMT = 69), providing only partial protection (2/4 survival). The prM–E 10 µg pooled sera (GMT = 514) were diluted to a range from 17 to 23 (GMT = 19) and failed to protect mice. These data, together with the increase in nAb titers on day 21, may indicate an active viral replication and subsequent nAb production. Furthermore, a vaccination study using BALB/c mice showed the effectively balanced induction of IgG1 and IgG2a immunoglobulin isotypes after vaccination with 5 µg of ZIKV prM–E mRNA-LNP. Moreover, activated CD4^+^ and CD8^+^ T-cells produced IFN-γ and TNF-α. All things considered, the protection seen in vaccinated AG129 mice might have been a combination of nAbs and cytotoxic T-cells, while the passive immunization of serum was not sufficient to protect the mice because of the lack of cytotoxic T-cells needed for viral clearance.

mRNA vaccine technologies are flexible vaccine platforms capable of synthesizing mRNAs that encode for various protein antigens, along with fast, simplified, and unified protocols for vaccine production. These key features make them attractive for pandemic preparedness and the fight of ongoing pandemics, as highlighted by the current COVID-19. With at least 13 Zika vaccine candidates currently evaluated in phase 1 clinical trials [10], new challenges have arisen to continue vaccine development such as the current low endemicity of ZIKV, lack of funding, and the imperative to protect pregnant women and their unborn babies. Here, we described a new mRNA-based Zika vaccine candidate that follows a similar approach and validates other Zika mRNA vaccine candidates previously described [8,9,17,18,19,20,21]. As observed in prior studies [8,18,19], mRNA vaccines induced high levels of neutralizing antibodies that protected BALB/c and C57BL/6 mice from ZIKV infection. The survival protection was assessed by treating mice with an antibody to disrupt type I IFN signaling after the challenge, making the animals transiently susceptible to ZIKV pathogenesis [22]. In the present study, we used genetically engineered mice that lack the IFN type I (IFN-α/β) and II (IFN-γ) receptors.⁠ Therefore, the animals were highly susceptible to ZIKV infection, making this model appropriate to assess the protection given by our vaccine. Furthermore, mRNA vaccines induce innate, humoral, and cellular immune responses [23,24,25].

While we did not observe any adverse effects after mRNA vaccination, future preclinical studies are needed for a full toxicology study. It is also important to evaluate the vaccine’s efficacy in the nonhuman primate model because of the close phylogenetic relationship and physiological similarity to humans. Indian rhesus macaques (*Macaca mulatta*) can be vaccinated and challenged with ZIKV, showing similar kinetics of ZIKV infection to humans, and might be part of a future development [26]. Furthermore, the longevity of the antibody response and the role of T-cell-mediated immunity induced by this mRNA vaccine needs to be determined in a nonhuman primate model. In summary, our mRNA vaccine platform can be manipulated to encode any protein, including other arboviruses such as dengue virus, yellow fever virus, or chikungunya virus. Lastly, we emphasize the imminent potential risk of ZIKV to cause large-scale epidemics, for which we need the combined efforts of the scientific community.

## 5. Patents

B.P. and E.J. are inventors on various patents covering a variety of mRNA technology aspects. 

## Data Availability

The authors declare that the data supporting the findings of this study are available within the paper and supplementary materials. Further information is available from the corresponding authors upon reasonable request.

## Supplementary Materials

The following are available online at https://www.mdpi.com/article/10.3390/vaccines9121464/s1, Raw data for Figure 2, Figure 3 and Figure 4.
